# Patterns of *Flavivirus* Seroprevalence in the Human Population of Northern Laos

**DOI:** 10.4269/ajtmh.15-0072

**Published:** 2015-11-04

**Authors:** James V. Conlan, Khamphouth Vongxay, Boualam Khamlome, Richard G. Jarman, Robert V. Gibbons, Stanley G. Fenwick, R. C. A. Thompson, Stuart D. Blacksell

**Affiliations:** School of Veterinary and Life Sciences, Murdoch University, Murdoch, Western Australia; Division of Veterinary Services, Department of Livestock and Fisheries, Ministry of Agriculture and Forestry, Vientiane, Lao People's Democratic Republic; Department of Communicable Diseases Control, Ministry of Health, Vientiane, Lao People's Democratic Republic; United States Army Medical Component, Armed Forces Research Institute of Medical Sciences, Bangkok, Thailand; Mahidol-Oxford Tropical Medicine Research Unit, Faculty of Tropical Medicine, Mahidol University, Bangkok, Thailand; Centre for Tropical Medicine and Global Health, Nuffield Department of Clinical Medicine, Churchill Hospital, Oxford, United Kingdom

## Abstract

A total of 1,136 samples from 289 households in four provinces in northern Laos were subjected to Japanese encephalitis virus (JEV) and dengue virus hemagglutination inhibition (DENV HI). Overall, antibodies to JEV were detected by HI in 620 (54.6%) of 1,136 people; of which 217 (19.1%) had HI activity against JEV only. Antibodies to DENV4 were detected by HI in 526 (46.3%) of 1,136 people; of which 124 (10.9%) had HI activity against DENV4 only. Antibodies to DENV1–3 were detected by HI in 296 (26.1%), 274 (24.1%), and 283 (24.9) of 1,136 people, respectively; of which 7, 1, and 0, respectively, had HI activity against DENV1–3 only. JEV was the most prevalent *Flavivirus* in Oudomxay, Luangprabang, and Huaphan provinces and DENV4 was the most prevalent in Xiengkhouang province. Seroprevalence for JEV increased with increasing age and wealth and was higher in villages where rice was cultivated in paddy fields and highest for people of Lao-Tai ethnicity.

## Introduction

Japanese encephalitis virus (JEV) and dengue virus (DENV) are major causes of death and disability in southeast (SE) Asia.^1^,^2^ Both JEV and DENV are endemic in Laos with reoccurring epidemics during the monsoonal wet season.^2^–^4^ A large proportion of the Lao population live within the vicinity of rice paddy and pig production,^5^,^6^ ensuring suitable conditions for JEV transmission from pigs to humans. Japanese encephalitis incidence has reportedly increased in recent years,^7^ even though there are no reliable estimates of incidence due to underreporting.^1^,^8^ Nevertheless, Japanese encephalitis infection in high-incidence countries that do not practice mass vaccination (such as Laos) has been estimated to be 10.6 cases per 100,000 population for children under 15 years and 0.7 cases per 100,000 population for those aged 15 years and older.^8^ For the period from 2006 to 2012, the annual notification rate for dengue ranged from 62 to 367 cases per 100,000 population, with the peak period of transmission from May to October.^4^

The epidemiology of JEV and DENV in the mountainous and ethnically diverse regions in northern Laos remains largely unknown. The principal aim of this study was to determine the spatial and age-related distribution of *Flavivirus* seroprevalence in northern Laos, including the influence rice paddy cultivation has on seroprevalence. We report the results of a cross-sectional study in four northern provinces of Laos and discuss the results in the context of potential mass vaccination to prevent JEV infection in the human population.

## Materials and Methods

### Ethics statement.

Written informed consent was obtained from all participants aged 15 years and older and from the parents or legal guardians of children < 15 years of age. The study protocol was reviewed and approved by the Murdoch University Human Ethics Committee (Project no. 2008/266) and the Lao Ministry of Health National Ethics Committee for Health Research (no. 239/NECHR) before commencing this study.

### Study design and questionnaire.

The sera used in this survey were also used in already published surveys of the seroprevalence of parasitic diseases such as cysticercosis and trichinellosis. The survey design, sample size calculations, and methodology have been described in detail elsewhere.^9^ The sample size calculations were based on estimates of taeniasis prevalence in the target populations.^9^ In brief, Laos is an ethnically diverse country with 49 ethnic groups classified into four ethnolinguistic families, Lao–Tai, Mon–Khmer, Hmong–Mien, and Sino–Tibetan.^10^ The study was conducted in four provinces in northern Laos, Oudomxay, Luangprabang, Huaphan, and Xiengkhouang. One district in each province, Xay, Xiengngeun, Viengxay, and Pek districts, respectively, was randomly selected for inclusion in the survey. To minimize disruption to seasonal labor demands and maximize participation, the survey was conducted in the dry season from January to March 2009. Fourteen households were randomly selected in each village and all household members ≥ 6 years of age were asked to participate. A venous blood sample of 2–3 mL was collected and the serum fraction was stored at −20°C. A household questionnaire was administered to the head of each household with his/her family present to determine age and ethnicity and assess house characteristics and assets owned. Rice paddy cultivation was determined during a preceding scoping survey to characterize the villages participating in the study.

### Serological analysis.

Serum samples were pretreated with acetone, and the ability of test serum antibodies to inhibit JEV and DENV 1–4 sucrose–acetone–extracted mouse brain antigens agglutinating goose erythrocytes was assayed by using a microtiter adaptation of the method of Clarke and Casals^11^ with an initial dilution of 1:10. Serum samples were serially diluted to 1:10,240 and hemagglutination inhibition (HI) titers > 10 were considered positive for JEV or DENV antibody. All samples were tested for JEV and the four dengue viruses (DEN1–DEN4) serotypes.

### Data analysis.

Seroprevalence was calculated as the proportion of serum samples with a positive HI titer in the sampled population. Samples were scored as either JEV or DEN1–DEN4 if they produced a 2-fold higher titer to the homologous viruses or scored as multiple previous *Flavivirus* infections if samples were positive for DENV and JEV without a 2-fold higher titer.^12^

The questionnaire and laboratory test data were entered into a spreadsheet (Excel; Microsoft, Redmond, WA) and subsequent analysis was carried out in STATA/IC version 10 (Stata Corp LP, College Station, TX). The socioeconomic status of each household was calculated by using principal component analysis of household assets as described previously.^13^ The households were ranked into wealth quintiles according to their cumulative standardized asset scores. Univariate logistic regression without adjustment was used to test associations between serological status and gender, location, ethnicity, age, wealth status, and rice paddy cultivation. The final analysis only considered persons with serologic and questionnaire data.

## Results

A total of 1,582 persons in 332 households were eligible to participate in this survey. Of these, 1,419 (89.7%) individuals from 324 households, aged 6–91 years, provided a blood sample and a completed questionnaire. After testing for multiple pathogens before this study, there was limited serum remaining for the JEV and DENV HI assays and in total 1,136 samples from 289 households were subjected to JEV and DENV HI and had a completed questionnaire. Antibodies to any of the five flaviviruses tested were detected in 765 of 1,136 people (67.3%; 95% confidence interval [CI]: 64.6–70.1%) (Table 1), of which, HI titers of 10, 20, and 40 were most commonly observed for all viruses (Table 2) and cross-reactivity was common. Antibodies to all five flaviviruses were detected by HI in 240 (21.1%) of 1,136 people assessed and antibodies to JEV were detected by HI in 620 (54.6%) of 1,136 people; of which 217 (19.1%) had HI activity against JEV only. Antibodies to DENV4 were detected by HI in 526 (46.3%) of 1,136 people; of which 124 (10.9%) had HI activity against DENV4 only. Antibodies to DENV1–3 were detected by HI in 296 (26.1%), 274 (24.1%), and 283 (24.9) of 1,136 people, respectively; of which 7, 1, and 0, respectively, had HI activity against DENV1–3 only.

Analysis of JEV or DENV1–DENV4 seroprevalence compared with variables including province, age, wealth status, ethnicity, and rice paddy cultivation revealed that JEV was the most prevalent *Flavivirus* in Oudomxay, Luangprabang, and Huaphan provinces and DENV4 was the most prevalent in Xiengkhouang province (Table 1). Seroprevalence for JEV increased with increasing age and wealth and was higher in villages where rice was cultivated in paddy fields and highest for people of Lao–Tai ethnicity. Conversely, DENV4 seroprevalence was highest in the youngest cohort of 6–11 year olds and in people of Hmong–Mien ethnicity. Seroprevalence of DENV1–3 was low in all provinces and across all variables assessed (Table 1).

**Figure 1.**
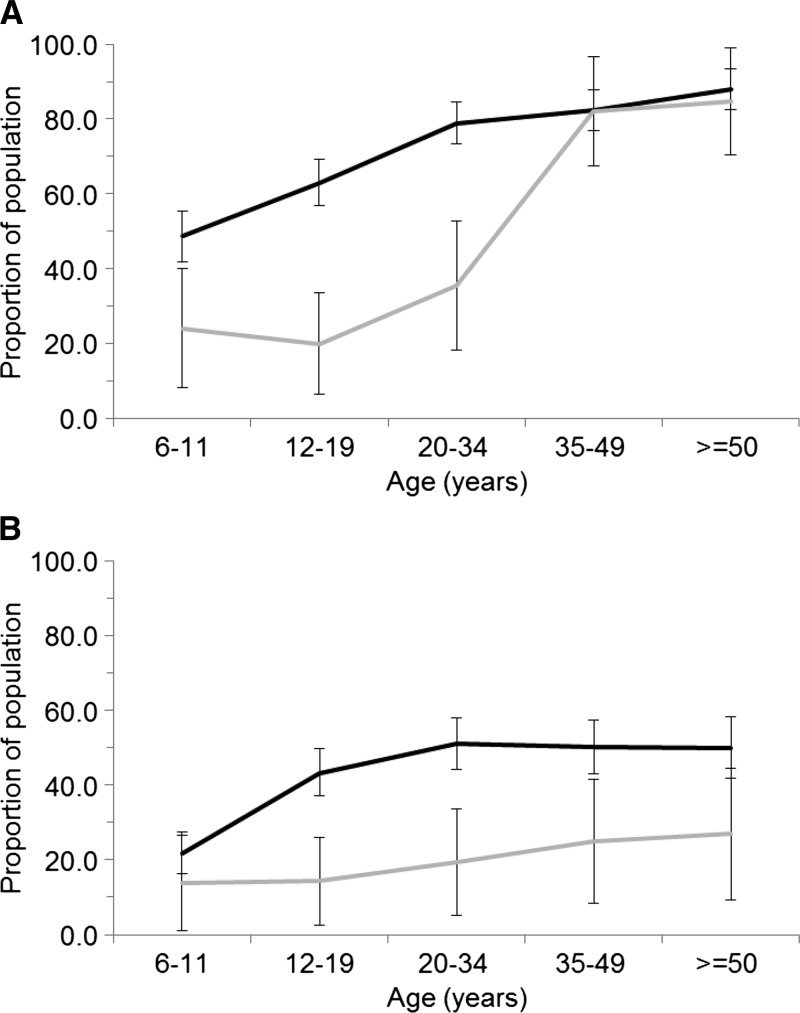
Sero-prevalence of hemagglutination inhibition (HI) antibodies against any of the tested flaviviruses (**A**) and Japanese encephalitis virus (JEV) (**B**) stratified by age. Black line represents those individuals living in villages cultivating paddy rice. Grey line represents those individuals living in villages not cultivating paddy rice. Error bars indicate 95% confidence interval (CI) of the prevalence.

The seroprevalence of HI antibodies to any of the flaviviruses tested was substantially higher in people living in the vicinity of rice paddy fields up to 34 years of age and was nearly identical for people aged 35 years and older (Figure 1A

). The seroprevalence of HI antibodies to JEV, on the other hand, was substantially higher in all age groups for people living in the vicinity of rice paddy fields (Figure 1B).

## Discussion

This study has revealed high levels of *Flavivirus* seroprevalence in northern Laos, with the majority due to previous infection with JEV. JEV is transmitted by paddy-breeding *Culex* mosquitoes, primarily *Culex tritaeniorhynchus*, in a zoonotic cycle involving ardeid wading birds (herons and egrets), pigs, and humans.^14^ DENV on the other hand is transmitted predominantly by *Aedes aegypti* mosquitoes that tend to proliferate in urban and semi-urban environments in SE Asia where suitable breeding sites such as man-made containers are common.^4^ Vector control is not practiced in Laos and the close proximity of water birds, pigs, human habitation, and rice cultivation means that vaccination is the only realistic control measure available.

Results presented here are similar to that of Vallée and others^15^ that reported a 57.7% JEV seroprevalence in urbanized Vientiane City. In that study, *Flavivirus* seroprevalence (both DENV and JEV) increased with age, with 84.6% positivity for people ≥ 35 years and 9.4% for children ≤ 6 years old.^15^ In Khammouane Province, a similar age-seroprevalence trend was observed where children < 5 years had a seroprevalence of 20%, increasing to > 50% for those older than 15 years of age.^12^ A hospital-based survey that examined causes of non-malarial fever in Laos found JEV seroprevalence of 7% in Luang Namtha (north) and 4% in Salavan (south) province.^3^

Dengue predominantly occurs in urban and peri-urban environments in Laos.^4^ The results presented here demonstrated dengue seroprevalence rates between 0.8% and 13.6% per serotype with DENV4 being dominant; there was notable difference between the DENV4 seroprevalence for Xiengkhouang province (42.5%) when compared with the other provinces. Interestingly, an examination of recent dengue outbreaks in Lao^4^ found that the four provinces that participated in this study (Oudomxay, Luangprabang, Huaphan, and Xiengkhouang) had relatively low transmission rates of 0–199 per 100,000 people relative to central and southern provinces, and it is not clear what has caused the increase in DENV4 seroprevalence in this case.

There is an increased risk of JEV transmission in a number of areas in Laos where smallholder pig husbandry, rice production systems, and poverty coexist. Pigs are recognized as a major risk factor and the source of JEV infection.^16^,^17^ The JEV seroprevalence in humans described in this study is consistent with JEV seroprevalences previously reported in pig populations of the same northern Lao provinces.^6^ JEV seroprevalence in pigs in northern Laos ranged from 59.8% (Luangprabang) to 90.2% (Oudomxay),^6^ which highlights the zoonotic risk posed by close proximity to village pig raising, or to larger-scale pig production. The JEV seroprevalence in humans more than doubled in those who tended a rice paddy from 19.4% to 42.4%, highlighting the epidemiological nexus.

This study has some methodological limitations. Inherent serological cross-reaction between flaviviruses requires results to be interpreted with caution. Diagnostic cutoffs and the interval between endpoint titers to discriminate DENV serotypes and JEV can affect the overall seroprevalence results and here we have chosen a 2-fold difference in endpoint titers limit to discriminate between flaviviruses, which is the same as a similar study conducted in southern Laos.^12^ The HI assay was used for the serological assessments as it is a simple technique that is cost effective and allows the processing of a large number of samples reasonably quickly without the need for complex laboratory infrastructure. The virus neutralization assay or plaque reduction neutralization test are more specific assays but require significant laboratory infrastructure such as the use of cell cultures, live virus, and in the case of JEV, a biosafety level three containment laboratory that was unavailable during the study. The use of enzyme-linked immunosorbent assay is another alternative, although these tests cannot discriminate between DENV serotypes and suffer significant cross-reaction problems with JEV. Another limitation was that travel information or vaccination information was not recorded for this cohort. However, given that the participants are village-based subsistence farmers, it might be reasonably assumed that they do not travel significant distances thereby exposing themselves to other diseases. At the time of the sample collections, there was no JEV vaccination in Laos that may have caused confounding results in the seroprevalence studies.

Vaccination is the single-most important control measure to prevent JEV transmission, especially so in less developed countries where vector control and vaccination of pigs is not readily achievable.^7^ It may be worthwhile considering the use of insect-proof pens for pig raising; however, this is of enormous practical difficulty in most low-resource settings. An important limitation of vaccination in isolated rural communities is the need for multiple vaccinations to elicit a protective immune response.^18^ Laos has commenced vaccination against JEV in northern Laos for children under 15 years of age as part of a multi-antigen vaccination campaign that reached in the order of 900,000 children in early 2014.^19^ There will be a need for continued surveillance to monitor the epidemiological situation in northern Laos to measure the impact of the vaccination program and adjust the vaccination program and schedule where appropriate.

## Supplementary Material

Supplemental Datas.

## Figures and Tables

**Table 1:** Seroprevalence for JEV and DENV 1–4 by province, age, ethnicity, wealth status, and rice paddy cultivation*

Population characteristics	*N*	Any flavivirus	JEV	DENV1	DENV2	DENV3	DENV4	Mixed *Flavivirus* positivity
Total	1,136	67.3 (64.6, 70.1)	39.4 (36.6, 42.3)	2.2 (1.3, 3.1)	0.8 (0.2, 1.3)	0.8 (0.8, 1.3)	13.6 (11.6, 15.6)	10.6 (8.8, 12.4)
Province								
Oudomxay	357	50.1 (44.9, 55.3)	43.1 (38.0, 48.3)	2.5 (0.9, 4.2)	0.8 (0.0, 1.8)	0.3 (0.0, 0.8)	0.0 (0.0, 0.0)	3.4 (1.5, 5.2)
Luangprabang	237	72.6 (66.9, 78.3)	44.7 (38.4, 51.1)	3.8 (1.4, 6.2)	2.1 (0.3, 3.9)	0.8 (0.0, 2.0)	0.4 (0.0, 1.2)	20.7 (15.5, 25.8)
Huaphan	262	90.8 (87.3, 94.3)	64.1 (58.3, 69.9)	2.3 (0.5, 4.1)	0.0 (0.0, 0.0)	0.0 (0.0, 0.0)	12.9 (8.9, 17.1)	11.5 (7.6, 15.3)
Xiengkhouang	280	62.9 (57.2, 68.5)	7.1 (4.1, 10.2)	0.4 (0.0, 1.1)	0.4 (0.0, 1.1)	2.1 (0.4, 3.8)	42.5 (36.7, 48.3)	10.4 (6.8, 13.9)
Age								
6–11	245	45.7 (39.5, 52.0)	20.8 (15.7, 25.9)	0.8 (0.0, 1.9)	0.8 (0.0, 1.9)	0.0 (0.0, 0.0)	20.4 (15.3, 25.5)	2.9 (0.8, 4.9)
12–19	273	57.5 (51.6, 63.4)	39.6 (33.7, 45.4)	1.8 (0.2, 3.4)	0.0 (0.0, 0.0)	0.4 (0.0, 1.1)	12.8 (8.8, 16.8)	2.9 (0.9, 4.9)
20–34	235	73.2 (67.5, 78.9)	46.8 (40.4, 53.2)	2.6 (0.5, 4.6)	0.4 (0.0, 1.3)	04 (0.0, 1.3)	16.2 (11.4, 20.9)	6.8 (13.8, 24.3)
35–49	215	82.3 (77.2, 87.4)	47.0 (40.3, 53.7)	4.2 (1.5, 6.9)	1.4 (0.0, 2.9)	1.4 (0.0, 3.0)	9.3 (5.4, 13.2)	19.1 (13.8, 24.3)
≥ 50	168	87.5 (82.5, 92.5)	46.4 (38.9, 54.0)	1.8 (0.0, 3.8)	1.8 (0.0, 3.8)	2.4 (0.0, 4.7)	6.5 (2.8, 10.3)	28.6 (21.7, 35.4)
Ethnicity								
Lao-Tai	490	82.9 (79.5, 86.2)	48.6 (44.1, 53.0)	1.4 (0.03, 2.5)	1.0 (0.1, 1.9)	1.4 (0.4, 2.5)	16.5 (13.2, 19.8)	13.9 (10.8, 16.9)
Mon-Khmer	432	49.8 (45.0, 54.5)	36.8 (32.2, 41.4)	3.0 (1.4, 4.6)	0.9 (0.0, 1.8)	0.5 (0.0, 1.1)	0.2 (0.0, 0.7)	8.3 (5.7, 10.9)
Hmong-Mien	214	67.3 (61.0, 73.6)	23.8 (18.1, 29.6)	2.3 (0.3, 4.4)	0.0 (0.0, 0.0)	0.0 (0.0, 0.0)	33.6 (27.3, 40.0)	7.5 (3.9, 11.0)
Wealth status								
Most poor	221	45.2 (38.7, 51.8)	27.6 (21.7, 33.5)	1.8 (0.0, 3.6)	0.9 (0.0, 2.2)	0.5 (0.0, 1.3)	8.5 (4.9, 12.3)	5.9 (2.8, 9.0)
Very poor	233	59.2 (52.9, 65.6)	37.3 (31.1, 43.6)	1.7 (0.0, 3.4)	0.4 (0.0, 1.3)	0.4 (0.0, 1.3)	12.9 (8.6, 17.2)	6.4 (3.3, 9.6)
Poor	249	79.5 (74.5, 84.5)	48.2 (42.0, 54.4)	3.6 (1.3, 5.9)	0.4 (0.0, 1.2)	0.8 (0.0, 1.9)	16.9 (12.2, 21.5)	9.6 (6.0, 13.3)
Less poor	245	80.0 (74.9, 85.0)	41.6 (35.4, 47.8)	1.6 (0.0, 3.2)	0.4 (0.0, 1.2)	1.6 (0.0, 3.2)	21.2 (16.1, 26.4)	13.5 (9.2, 17.8)
Least poor	188	70.1 (64.2, 77.3)	41.5 (34.4, 48.6)	2.1 (0.1, 4.2)	2.1 (0.1, 4.2)	0.5 (0.0, 1.6)	5.9 (2.5, 9.2)	18.6 (13.0, 24.2)
Rice paddy								
No	149	47.0 (38.9, 55.0)	19.4 (13.1, 25.8)	4.7 (1.3, 8.1)	1.3 (0.0, 3.2)	0.7 (0.0, 2.0)	6.7 (2.7, 10.7)	14.1 (8.5, 19.7)
Yes	987	70.4 (67.6, 73.3)	42.4 (39.4, 45.5)	1.8 (1.0, 2.7)	0.7 (0.2, 1.2)	0.8 (0.3, 1.4)	14.6 (12.4, 16.8)	10.0 (8.2, 11.9)

CI = confidence interval; DENV = dengue virus; HI = hemagglutination inhibition; JEV = Japanese encephalitis virus.

95% CI are displayed in parentheses.

* Samples were considered positive if they produced an HI reactive titer > 1:10. All samples were tested for JEV and the four dengue viruses (DEN1–DEN4) serotypes. Any *Flavivirus* indicates a positive HI titer for at least one of the five flaviviruses tested. Samples were scored as either JEV or DEN1–DEN4 if they produced a 2-fold higher titer to the homologous viruses or scored as mixed *Flavivirus* positivity if samples were positive for DEN and JEV without a 2-fold higher titer.

**Table 2:** Reactivity of serum in the HI assay for JEV and DENV 1–4

HI titer	Number of samples (% of total)				
	JEV	DENV1	DENV2	DENV3	DENV4
< 1:10	516 (45.4)	840 (73.9)	862 (75.9)	853 (75.1)	610 (53.7)
1:10	95 (8.4)	80 (7.0)	85 (7.5)	83 (7.3)	162 (14.3)
1:20	149 (13.2)	63 (5.6)	61 (5.4)	47 (4.1)	146 (12.9)
1:40	108 (9.5)	68 (6.0)	52 (4.6)	58 (5.1)	103 (9.1)
1:80	52 (4.6)	49 (4.3)	40 (3.5)	37 (3.3)	44 (3.9)
1:160	31 (2.7)	24 (2.1)	21 (1.9)	39 (3.4)	48 (4.2)
1:320	1 (0.1)	6 (0.5)	10 (0.9)	11 (1.0)	15 (1.3)
1:640	13 (1.1)	4 (0.4)	2 (0.2)	5 (0.4)	5 (0.4)
1:1,280	1 (0.1)	0 (0.0)	2 (0.2)	1 (0.1)	1 (0.1)
1:2,560	0 (0.0)	2 (0.2)	1 (0.1)	0 (0.0)	0 (0.0)
1:5,120	1 (0.1)	0 (0.0)	0 (0.0)	2 (0.2)	1 (0.1)
1:10,240	1 (0.1)	0 (0.0)	0 (0.0)	0 (0.0)	1 (0.1)

DENV = dengue virus serotypes 1−4; HI = hemagglutination inhibition; JEV = Japanese encephalitis virus.
